# Evaluation of the immunoprotective effects of eight recombinant proteins from *Baylisascaris schroederi* in mice model

**DOI:** 10.1186/s13071-023-05886-y

**Published:** 2023-07-28

**Authors:** Lang Xiong, Ling Chen, Yanxin Chen, Nengxing Shen, Ruiqi Hua, Guangyou Yang

**Affiliations:** Department of Parasitology, College of Veterinary Medicine, Sichuan Agricultural University, Chengdu, Sichuan China

**Keywords:** Recombinant proteins, *Baylisascaris schroederi*, Giant panda, Immunoprotective effect, Mouse model

## Abstract

**Background:**

*Baylisascaris schroederi* is the most common and harmful intestinal parasitic nematode of giant pandas, causing ascariasis. Although drug deworming is the main measure to control ascariasis in captive giant pandas, prolonged and repeated use of deworming drugs might induce resistance in nematodes and drug residues in giant pandas. Therefore, developing a safe and effective vaccine might provide a novel strategy to prevent ascariasis in captive giant pandas.

**Methods:**

Four highly expressed secretome genes encoding excretory and secretory proteins of *B. schroederi*, including transthyretin-like protein 46 (*Bs*TLP), uncharacterized protein (*Bs*UP), hypothetical protein 1 (*Bs*HP1), and hypothetical protein 2 (*Bs*HP2) and four functional genes [(encoding Galectin (*Bs*GAL), glutathione S-transferase (*Bs*GST), fatty acid-binding protein (*Bs*FABP), and thioredoxin peroxidase (*Bs*TPX)] were identified based on genome and transcriptome databases of *B. schroederi* and used to construct recombinant proteins via prokaryotic expression. Kunming mice were vaccinated subcutaneously twice with the recombinant proteins (50 μg/mouse) mixed with Quil A adjuvant with a 2-week interval and then orally challenged with 3000 infective eggs. The immunoprotective effects of the eight recombinant proteins on mice were assessed comprehensively using surface lesion histology scores of the mouse liver and lung, larval worm reduction, serum antibody levels (IgG, IgE, IgA, IgG1, and IgG2a), and cytokine production [interferon gamma (IFN-γ), interleukin (IL)-2, IL-4, IL-5, and IL-10].

**Results:**

Mice vaccinated with recombinant (r)*Bs*UP (76.5%), r*Bs*GAL (74.7%), and r*Bs*HP2 (71.5%) showed a significant (*P* < 0.001) reduction in the larval worm rate compared with that in the adjuvant control. Besides, the surface lesions in the liver and lung of the vaccinated mice were alleviated. Serum levels of total IgG, IgE, IgA, IgG1, IgG2a, and cytokines, including IL-10, IL-5, and IFN-γ, were significantly higher (*P* < 0.001) than those in the control group.

**Conclusions:**

The results showed that candidate three vaccines (r*Bs*UP, r*Bs*GAL, and r*Bs*HP2) could provide effective protection against egg infection in mice associated with a mixed Th1/2-type immune response.

**Graphical Abstract:**

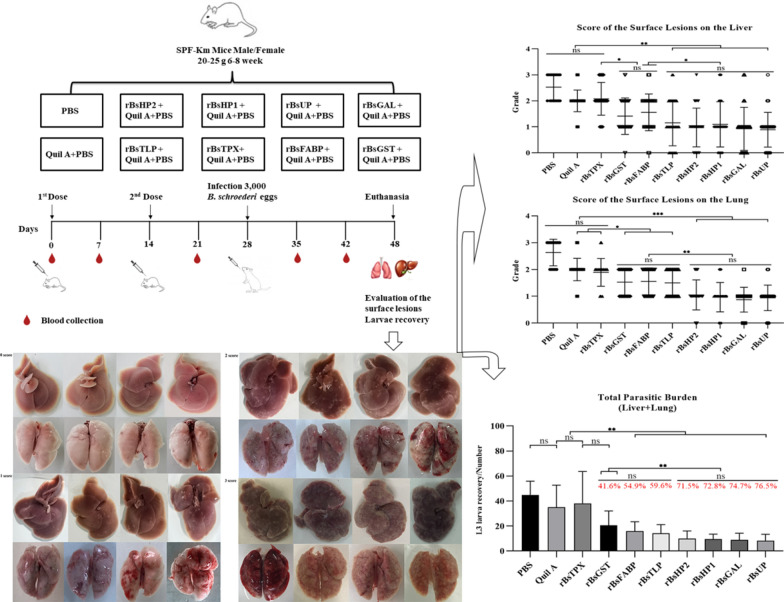

## Background

The giant panda (*Ailuropoda melanoleuca*) is a living fossil in the animal kingdom and is a world-famous rare animal. According to the 4th Giant Panda survey in China [1], the wild population comprises only 1864 giant pandas, and there were 699 captive giant pandas in 2022. *Baylisascaris schroederi* is the most common and harmful type of intestinal parasitic nematode in giant pandas [2], which can cause ascariasis, with symptoms of malnutrition, wasting, anemia, anorexia, and intestinal dysfunction. Larval migration can also lead to severe liver, lung, bile duct, and pancreatic lesions and even death of giant pandas [3]. This roundworm lays many eggs, which have strong viability in the environment, often causing repeated infection of hosts [4]. The infection rate of *B. schroederi* in wild pandas can be as high as 100%, and that of captive pandas can reach > 25% [5]. Anthelmintics (ivermectin, albendazole, and pyrantel pamoate) are the most important means of controlling ascariasis in captive giant pandas [6]; however, their repeated and long-term use can lead to nematode drug resistance [7]. Therefore, new methods have been sought urgently to prevent and control giant panda ascariasis, including developing an ascariasis vaccine. Following the infection of mice with infective eggs of *B. schroederi*, larvae hatched and penetrated the intestinal wall to migrate to the liver via the portal vein system. By day 7, the larvae left the intestine, and the second-stage larval counts peaked in the liver. Some larvae entered the lungs through the pulmonary artery, while only a few migrated to other organs such as the kidney and brain. By day 18, the larvae developed into third-stage larvae by the second molt in the liver. The third-stage larvae primarily parasitize the liver and can survive in the liver for over a year but not more than 2 months in the lungs [8]. *B. schroederi* differs from *Toxocara canis* and *Baylisascaris procyonis* because it does not accumulate in the muscles or nervous systems of mice and does not cause severe neurological diseases [9–11]. Similar to *Ascaris suum*, *Ascaris lumbricoides*, and *T. canis* infected-mouse models, *B. schroederi* could only develop into third-stage larvae in mice [12–15]. The mouse model has been proven to be an important and valid tool for screening candidate vaccines against *B. schroederi*. Four immunodominant antigens (Ag1, Ag2, Ag3, and PYP) were identified in the sera of *B. schroederi*-infected mice and induced partial immune protection in a mouse model by vaccination [13, 16–18]; however, no commercial vaccines are currently available.

Excretory/secretory proteins (ESPs) are active molecules directly released by parasites into host body fluids and tissues, playing a pivotal role in parasite infection, parasitism, immune evasion, and host immune regulation [19]. ESPs can directly interact with the host immune system to trigger antigen–antibody responses; therefore, ESPs have been regarded as a source of ideal candidate vaccines and immunodiagnostic antigens [20]. The advancement of omics technology has promoted the study of numerous parasites ESPs. These studies have demonstrated that ESPs possess the potential for protective immunity, which has accelerated the development of parasite vaccines [21]. Recently, 1639 secretome genes were predicted and analyzed using a bioinformatics pipeline based on the genome and transcriptome data of *B. schroederi* [22]. In the present study, four highly expressed secretome genes encoding ESPs [transthyretin-like protein 46 (*Bs*TLP), uncharacterized protein (*Bs*UP), hypothetical protein 1 (*Bs*HP1), and hypothetical protein 2 (*Bs*HP2)] and four functional genes [encoding galectin (*Bs*GAL), glutathione S-transferase (*Bs*GST), fatty acid-binding protein (*Bs*FABP), and thioredoxin peroxidase (*Bs*TPX)] showing the potential of early diagnosis and candidate vaccines of *B. schroederi* were selected and expressed via prokaryotic expression system [23–29]. The immunoprotective effects of the eight recombinant proteins in mice against infection by *B. schroederi* were evaluated by worm burdens, liver and lung surface lesion histology scores, humoral immune response, and cellular immune response. Our study provides potential candidate vaccines to prevent and control panda ascariasis.

## Methods

### Parasites

Fresh adult worms and eggs of *B. schroederi* were collected from infected giant pandas in the Chengdu Research Base of Giant Panda Breeding (Sichuan, China). The eggs were filtered and screened for impurities and then cultured in a mixture of 0.9% normal saline and 2.5% formalin until most developed into the embryonated infective stage.

### Experimental model

Male and female Kunming mice (*n* = 360; 6–8 weeks old; 20–25 g) were purchased from Chengdu Dossy Experimental Animals Co., Ltd. (Sichuan, China). All mice were acclimatized for 1 week, provided free access to water and food, and strictly housed in an SPF environment (SPF mice).

### Expression and purification of eight recombinant proteins from *B. schroederi*

Total RNA extraction from adult worms was conducted using a TianGen RNA extraction kit according to the manufacturer's instructions (TianGen, Beijing, China). RNA reverse transcription was performed using a Thermo First Strand cDNA Synthesis Kit (Thermo Fisher Scientific, Waltham, MA, USA) and oligonucleotide (DT 18) primers.

Signal peptides and cleavage sites of TPX, GST, FABP, TLP, HP2, HP1, GAL, and UP were predicted using SignalP 3.0 (http://www.cbs.dtu.dk/services/SignalP/), and the parts encoding the signal peptides were excluded. B cell epitopes (http://tools.iedb.org/bcell/), antigen toxicity (https://webs.iiitd.edu.in/raghava/toxinpred2/), and antigen allergy (https://webs.iiitd.edu.in/raghava/algpred2/) were predicted for all antigens. DNA fragments of all genes obtained by PCR amplification were subcloned in the prokaryotic expression vector pET32a. The recombinant vectors were transformed into *Escherichia coli* BL21 (DE3)/Rosetta cells. Verified transformants were cultured for 16 h at 24 °C after induction with 0.5 mM isopropyl β-d-1-thiogalactopyranoside. Cells were harvested by centrifugation (8000 ×*g*, 15 min) and resuspended in pH7.4 lysis buffer (Tris–HCl, Solarbio, Beijing, China) before sonication. The soluble supernatant containing the target protein was collected by centrifugation (10,000 ×*g*, 4 °C, 15 min) and identified by SDS-PAGE. Recombinant proteins were purified using nickel column affinity chromatography (Histrap™ HP column; GE Healthcare, Amersham, UK) by elution buffer (10 mM Na_2_HPO_4_, 10 mM NaH_2_PO_4_, and 500 mM imidazole) and diluted in PBS overnight at 4 °C [30]. All purified proteins were separated using 12% sodium dodecyl sulfate–polyacrylamide gel electrophoresis. The protein concentration was measured at A280 using a NanoDrop spectrophotometer (Nanodrop Technologies, Wilmington, DE, USA).

### Western blotting analysis

All recombinant proteins were resolved using 12% sodium dodecyl sulfate–polyacrylamide gel electrophoresis, followed by transfer onto polyvinylidene fluoride membranes (0.45 μm, Bio-Rad, Hercules, CA, USA). The membranes were washed using phosphate-buffered saline-Tween 20 (PBST), blocked using 5% skimmed milk in PBST at 37 °C for 2 h, and incubated with a pool of sera from mice infected with *B. schroederi* (non-infected mice sera as controls) as the primary antibodies at 4 °C overnight. The next day, the membranes were treated with enzyme-labeled goat anti-mouse IgG (Abclonal, Wuhan, China) secondary antibody for 1 h at 37 °C. The immunoreactive protein bands were visualized using the enhanced chemiluminescent (ECL) reagent (Beyotime, Shanghai, China).

### Immunization, challenge infection, and sera collection

The experimental group consisted of two control groups [PBS and adjuvant (*n* = 20 each group)] and eight vaccine groups (*n* = 20 for each group and *n* = 20 for another repeat group). Mice in the eight vaccine groups were immunized twice subcutaneously at 14-day intervals with 50 μg of recombinant proteins individually mixed 1:1 with Quil A (Sigma, St. Louis, MO, USA) adjuvant (total volume of 200 μl). PBS + Quil A and PBS were injected into the control groups using the same methods (Table 1).

**Table 1:** Grouping of mouse immune protection experiments

Groups	Number	Immunized-0/14 (dpv)	Blood collection (dpv)	Challenge (dpv)	Recovery (dpc)
TPX	20♂/20♀	r*Bs*TPX + Quil A + PBS	0/7/21/35/42	14	20
GST	20♂/20♀	r*Bs*GST + Quil A + PBS	0/7/21/35/42	14	20
FABP	20♂/20♀	r*Bs*FABP + Quil A + PBS	0/7/21/35/42	14	20
TLP	20♂/20♀	r*Bs*TLP + Quil A + PBS	0/7/21/35/42	14	20
HP2	20♂/20♀	r*Bs*HP2 + Quil A + PBS	0/7/21/35/42	14	20
HP1	20♂/20♀	r*Bs*HP1 + Quil A + PBS	0/7/21/35/42	14	20
GAL	20♂/20♀	r*Bs*GAL + Quil A + PBS	0/7/21/35/42	14	20
UP	20♂/20♀	r*Bs*UP + Quil A + PBS	0/7/21/35/42	14	20
Adjuvant	10♂/10♀	Quil A + PBS	0/7/21/35/42	14	20
Blank	10♂/10♀	PBS	0/7/21/35/42	14	20

*dpv* days post-vaccination, *dpc* days post-challenge

**Fig. 1:**
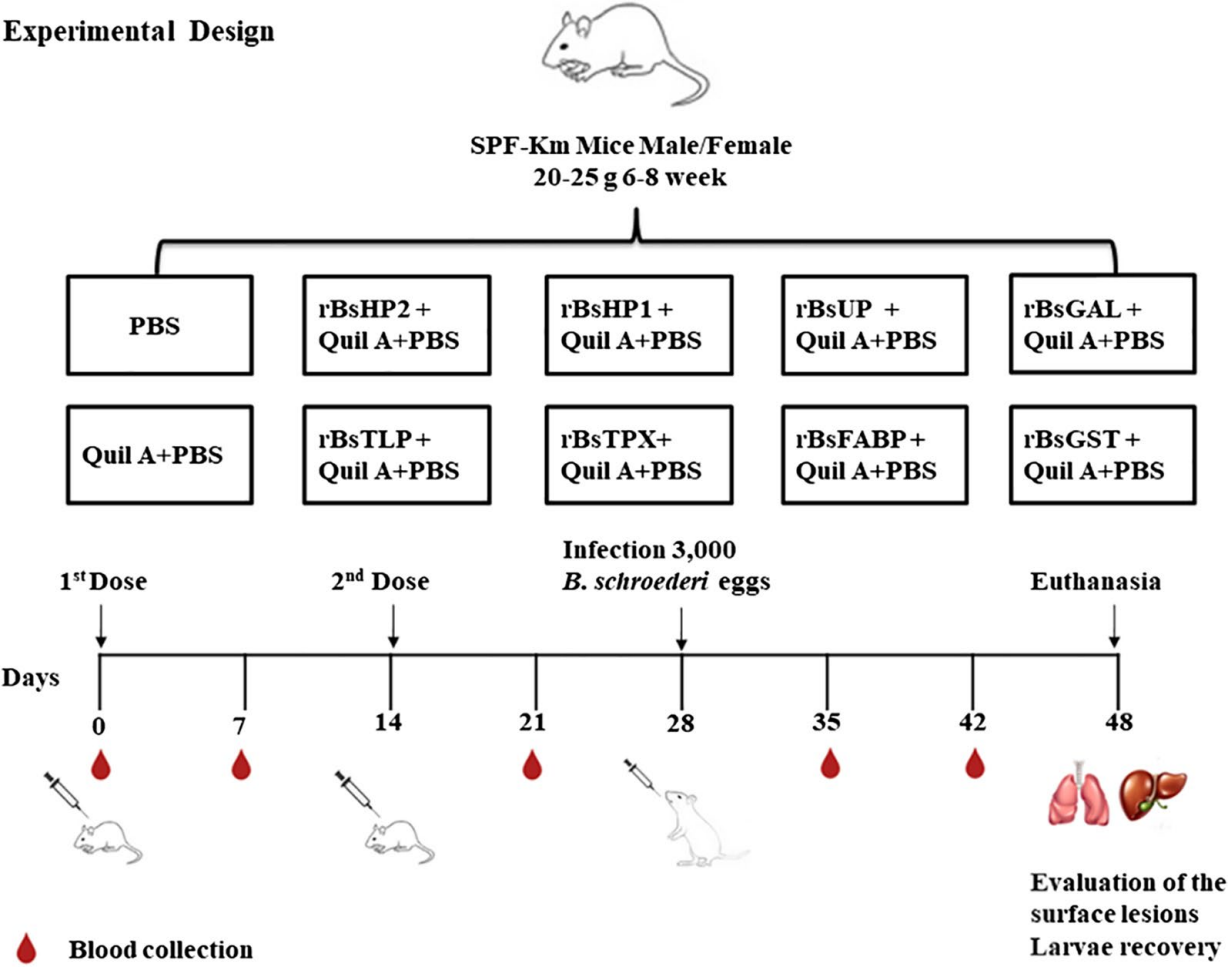
Schematic showing the experimental design of immune protection in mice

The mice were administered 3000 embryonated *B. schroederi* eggs via oral gavage at 14 days after the second immunization [31]. Serum was collected from the mice at five stages (Time 0–4), comprising before the primary immunization, 7 days after the primary immunization, 7 days after the second immunization, 7 days after challenge, and 14 days after challenge. Collected sera were stored at − 80 °C (Fig. 1).

### Evaluation of the surface lesions on the liver and lung of mice

All mice were killed at 20 days after infection. Their lungs and livers were removed, photographed, and weighed, followed by evaluation of surface lesions.

Evaluation indicators comprised:

Score 0: the liver appears light red with a smooth surface and lack spots; the lung appears pink and has a smooth surface, and soft and uniform texture;

Score 1: the liver shows scattered small white spots; the lung shows mild congestion, swelling, and atrophy;

Score 2: the liver shows scattered leaf spots; the lung shows moderate congestion, swelling, atrophy, and early transparent blister formation;

Score 3: the liver appears dark red with white plaques; the lung appears dark pink and coarse, with severe swelling, congestion, edema, and transparent blister formation.

### Recovery of larvae from livers and lungs

Twenty days after challenge, immune protection was evaluated by calculating the total number of larvae taken from the lungs and liver. Tissues (lungs and liver) were harvested and placed into PBS-filled Eppendorf tubes, finely minced using surgical scissors, and then incubated at 37 °C for 2 h in a modified Baermann apparatus [32]. The recovered larvae were fixed using 4% neutral formalin and counted under a microscope [33]. The percentage reduction in the parasite burden was calculated as follows: % reduction rate of larvae = [(average recovered larvae in adjuvant mice−average recovered larvae in vaccine mice)/average recovered larvae in adjuvant mice] × 100 [16].

### Specific antibody production detection by enzyme-linked immunosorbent assay (ELISA)

The levels of antigen-specific IgG (total, subclasses IgG1, IgG2a), IgE, and IgA in sera were measured using an indirect ELISA, according to the method of Gazzinelli Guimarães [34]. ELISA plates (Corning, NY, USA) were coated with 1 µg purified recombinant protein in a total volume of 100 µl per well overnight at 4 °C. For IgG detection, sera were used at a dilution of 1/200. Anti-IgG, IgG1, and IgG2a secondary antibodies (horseradish peroxidase-conjugated-Goat anti-Mouse; Abclonal) were added at dilutions of 1/3000. For IgE and IgA detection, sera were used at 1/20, and IgE and IgA secondary antibodies (horseradish peroxidase-conjugated-goat anti-mouse; Invitrogen, Waltham, MA, USA) diluted at 1/3000 and 1/1000 [15] were used, respectively. All assays were conducted in duplicate.

### Cytokine profiles

Sera from mice after the second immunization were analyzed for cytokine levels [interleukin (IL)-2, IL-4, IL-5, IL-10, and interferon gamma (IFN-γ)] using a flow cytometry-based BD Cytokine Bead Array (BD Biosciences, San Jose, CA, USA).

### Statistical analysis

All data were pretested using normality and log-normality tests (Kolmogorov-Smirnov normality test). Statistical significance was assessed using one-way analysis of variance (ANOVA; nonparametric or mixed) followed by Tukey's and Holm-Sidak’s multiple comparisons test (for parametric data) or a Kruskal-Wallis test with Dunn’s multiple comparisons test (for non-parametric data). The two-way ANOVA followed by Tukey’s multiple comparison tests was used to analyze multiple variables. For all tests, *P* ≤ 0.05 was considered significant. GraphPad Prism 8.0.2 (GraphPad Inc., La Jolla, CA, USA) was used to conduct the statistical analyses.

## Results

### Expression, purification, and antigenicity analysis of eight recombinant proteins from *B. schroederi*

The entire open reading frames of the *BsTPX* (GenBank accession number: OQ263159), *BsGST* (GenBank accession number: AJH66211.1), *BsFABP* (GenBank accession number: OQ263161), *BsTLP* (GenBank accession number: OP807116), *BsHP2* (GenBank accession number: OP807114), *BsHP1* (GenBank accession number: OP807113), *BsGAL* (GenBank accession number: OQ263160), and *BsUP* (GenBank accession number: OP807115) genes (588, 621, 438, 378, 198, 198, 429, and 225 bp, respectively) were amplified by PCR using cDNA as a template. After cloning and sequencing, the amplified nucleotide fragments were judged to be consistent with the transcriptome data and the entries in the NCBI database. Prediction results showed that the eight antigens were non-toxic and non-allergic, and the number of B cell antigen epitopes was between 2 and 11.

**Fig. 2:**
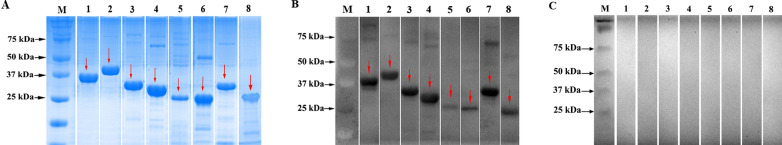
Purification of eight recombinant proteins. **A** After purification, the recombinant proteins were separated using sodium dodecyl sulfate-polyacrylamide gel electrophoresis. **B** Western blot showing that the sera from mice infected with *B. schroederi* recognized the eight recombinant proteins. **C** Western blots with non-infected mice sera. Lane: M: Protein molecular weight marker (kDa); 1, r*Bs*TPX; 2, r*Bs*GST; 3, r*Bs*FABP; 4, r*Bs*TLP; 5, r*Bs*HP2; 6, r*Bs*HP1; 7, r*Bs*GAL; 8, r*Bs*UP

The eight recombinant proteins were expressed in a soluble form after induction, with molecular weights of approximately 38, 40, 35, 31, 24, 24, 33, and 25 kDa, respectively (including a 17 kDa Trx-His-S tag from pET32a), consistent with the expected sizes of the predicted recombinant products (Fig. 2A).

The eight recombinant proteins reacted with the serum of *B. schroederi*-infected mice and produced signal bands at the corresponding positions. Besides, none of the cross-reaction bands were observed since these proteins incubated with sera from uninfected mice. The results revealed that the eight recombinant proteins had strong antigenicity (Fig. 2B, C).

### Larva reduction

**Fig. 3:**
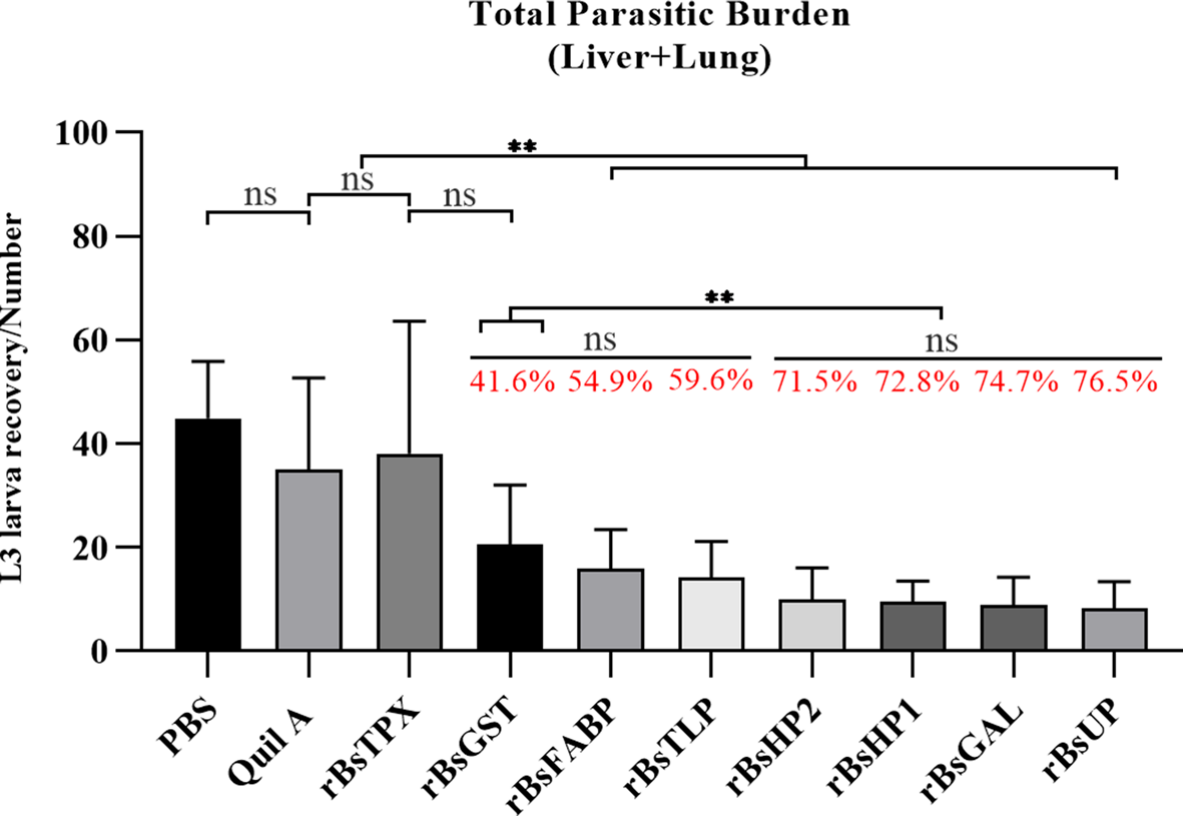
Reduction rate of larvae in mice immunized with the eight recombinant proteins. (Treatment with adjuvant only formed the control group; significance analysis: *ns* no significant difference, ^*^P < 0.05, ^**^P < 0.01, ^***^P < 0.001)

The total larva recovery from the liver and lung was significantly reduced in the mice in the r*Bs*UP (76.5% reduction, *P* < 0.001), r*Bs*GAL (74.7% reduction, *P* < 0.001), r*Bs*HP1 (72.8% reduction, *P* < 0.001), r*Bs*HP2 (71.5% reduction, *P* < 0.001), r*Bs*TLP (59.6% reduction, *P* < 0.001), and r*Bs*FABP (54.9% reduction, *P* = 0.003) groups compared with that in the adjuvant group. However, the total larva count from the liver and lung in the r*Bs*TPX and r*Bs*GST (41.6% reduction, *P* = 0.16) group was not significantly reduced. The total larva numbers of the rBsUP, rBsGAL, rBsHP1, and rBsHP2 groups were significantly reduced (*P* < 0.008) compared with those of the rBsGST and rBsTPX groups (Fig. 3).

### The comprehensive score of the surface lesions on the liver and lung

**Fig. 4:**
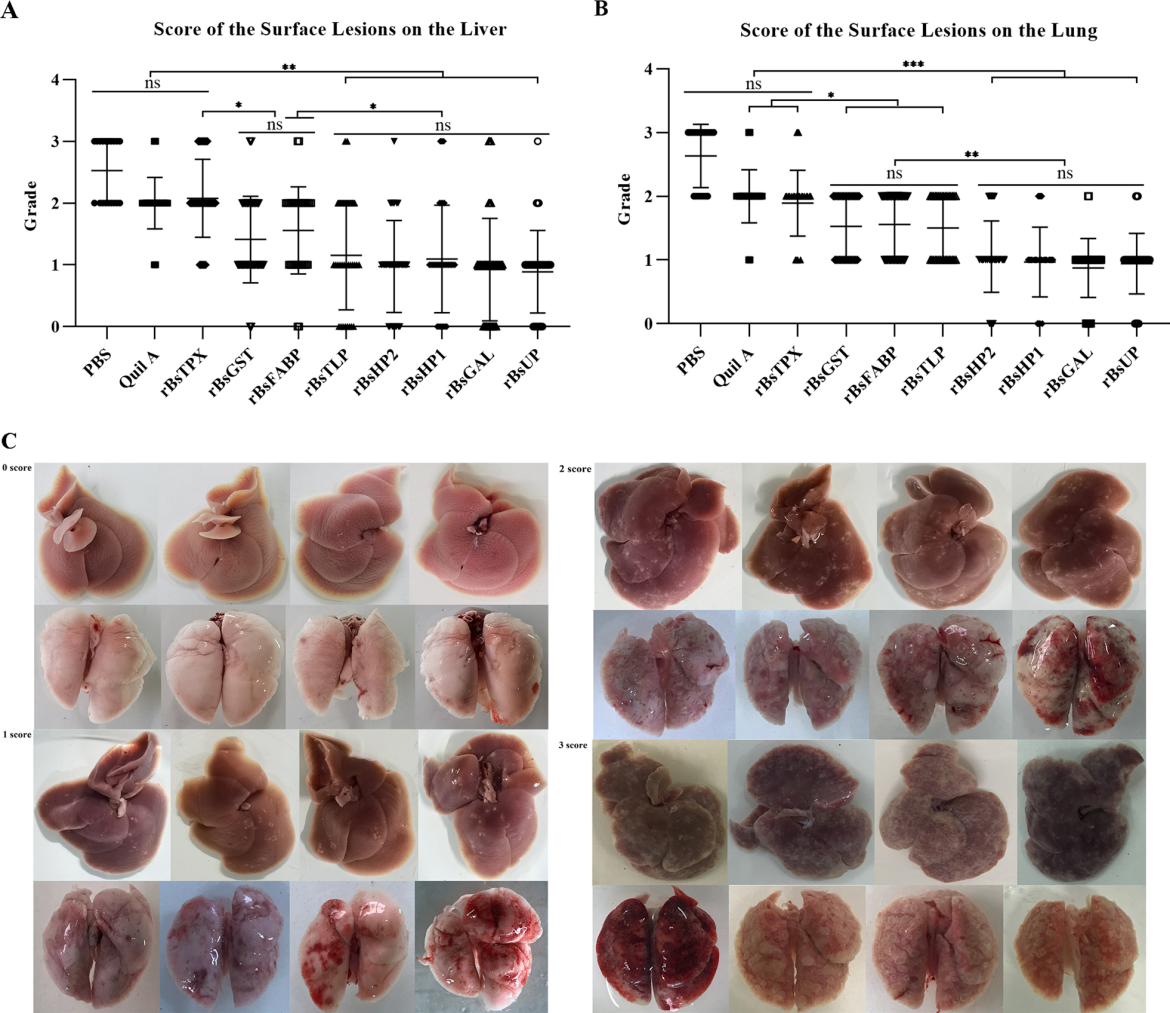
Comprehensive score of the surface lesions on the liver and lung in the different vaccine groups. **A** Score of the surface lesions on the liver, **B** score of the surface lesions on the lung, **C** scores and typical images of lung and liver surface lesions. (Treatment with adjuvant only formed the control group; score ± standard error of mean, significance analysis: *ns* no significant difference, ^*^P < 0.05, ^**^P < 0.01, ^***^P < 0.001)

The livers of mice in the PBS (score, 2.53 ± 0.12) and adjuvant (score, 2.00 ± 0.09) groups showed severe lesions such as dark red color and severe white plaques on the surface. Mice in vaccine groups r*Bs*HP2 (score, 0.97 ± 0.12, *P* < 0.001), r*Bs*HP1 (score, 1.10 ± 0.16, *P* < 0.001), r*Bs*UP (score, 0.89 ± 0.11, *P* < 0.001), r*Bs*GAL (score, 0.93 ± 0.13, *P* < 0.001), r*Bs*TLP (score, 1.16 ± 0.16, *P* < 0.001), r*Bs*GST (score, 1.41 ± 0.12,* P* = 0.004), and r*Bs*FABP (score, 1.56 ± 0.18, *P* = 0.03) showed significantly alleviated liver lesions on the surface, whereas the r*Bs*TPX group (score, 2.08 ± 0.10, *P* = 0.94) failed to relieve the liver lesions in mice (Fig. 4A).

Severe swelling, congestion, edema, and transparent blisters were observed in the lungs of mice in the PBS (score, 2.63 ± 0.11) and adjuvant (score, 2.0 ± 0.09) groups. Mice immunized with r*Bs*HP2 (score, 1.05 ± 0.09, *P* < 0.001), r*Bs*HP1 (score, 0.97 ± 0.10, *P* < 0.001), r*Bs*UP (score, 0.94 ± 0.08, *P* < 0.001), r*Bs*GAL (score, 0.88 ± 0.07, *P* < 0.001), r*Bs*TLP (score, 1.50 ± 0.09, *P* = 0.004), r*Bs*GST (score, 1.53 ± 0.09, *P* = 0.007), and r*Bs*FABP (score, 1.56 ± 0.09, *P* = 0.01) had significantly attenuated lung lesions. However, the lungs of mice immunized with r*Bs*TPX (score, 1.89 ± 0.08, *P* = 0.49) showed severe lesions (Fig. 4B). The scores and typical images of the lung and liver surface lesions are shown in Fig. 4C.

### Detection of the antigen-specific antibodies in sera

**Fig. 5:**
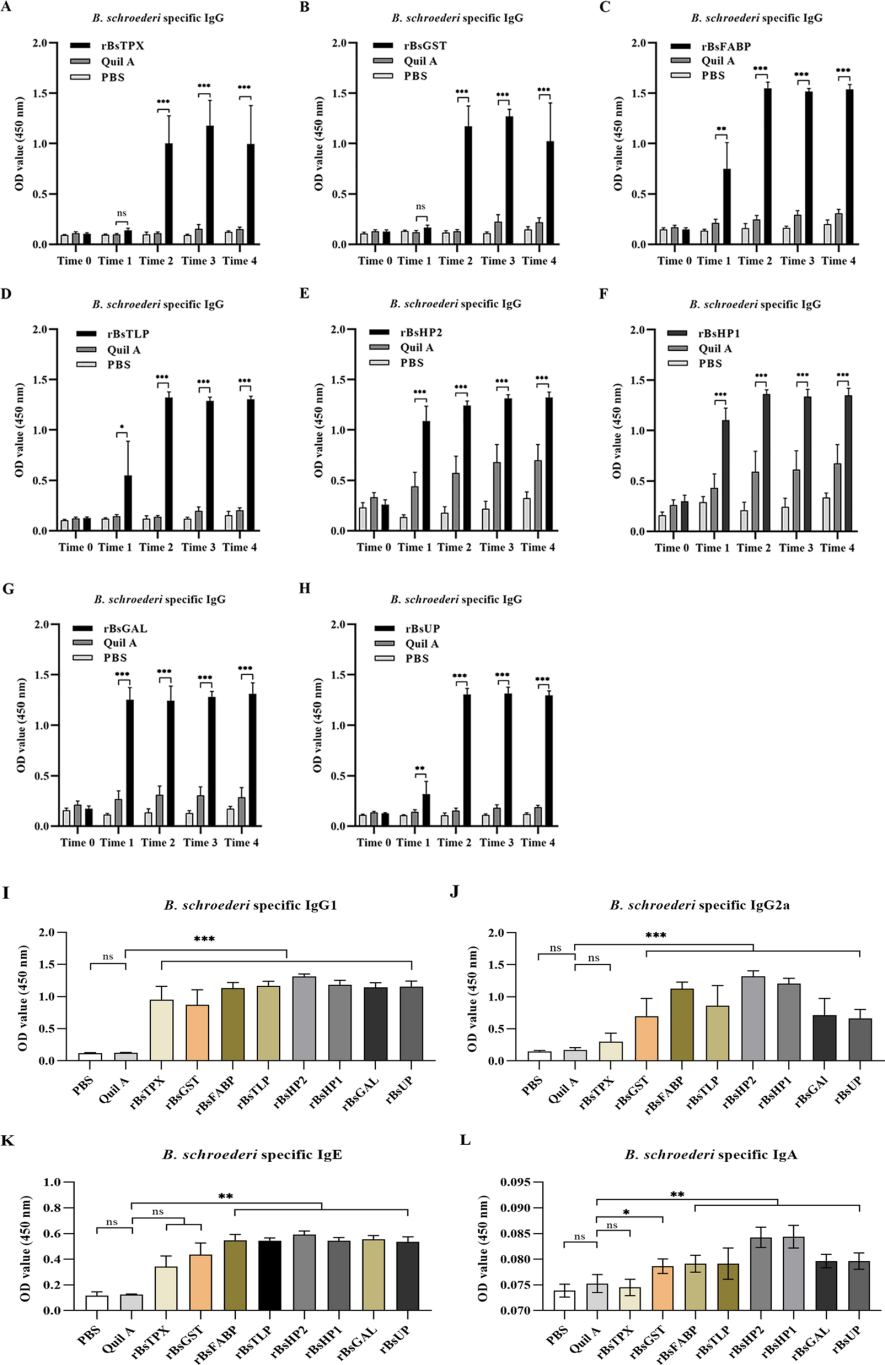
Results of specific antibody responses induced by *B. schroederi* antigens (r*Bs*TPX, r*Bs*GST, r*Bs*FABP, r*Bs*TLP, r*Bs*HP2, r*Bs*HP1, r*Bs*GAL, and r*Bs*UP) in mice. **A**–**H** *B. schroederi*-specific IgG before primary immunization (Time 0) at 7 days after the primary immunization (Time 1), 7 days after the second immunization (Time 2), 7 days after challenge (Time 3), and 14 days after challenge (Time 4). **I** IgG1, **J** IgG2a, **K** IgE, and **L** IgA in the sera of the mice at 7 days the after the second immunization (Time 2). (Treatment with adjuvant only formed the control group; significance analysis: *ns* no significant difference, ^*^P < 0.05, ^**^P < 0.01, ^***^P < 0.001)

The levels of antigen-specific IgG were low in all groups of sera before the primary immunization. After two immunizations, the IgG levels in all antigen groups were significantly elevated (*P* < 0.001) and then persisted at higher levels after parasite infection compared with the adjuvant group (Fig. 5A–H). The IgG subclasses in the sera of all groups of mice were analyzed. High IgG1 and IgG2a levels in sera were produced after the second immunization; the IgG1 (Fig. 5I) levels in all vaccine groups were elevated significantly (*P* < 0.001) compared with those in the adjuvant group, and IgG2a (Fig. 5J) also increased significantly (*P* < 0.001), except in the r*Bs*TPX group. The other antibody isotypes (IgE and IgA) in the sera of all groups of mice after the second immunization were further evaluated. The specific IgE levels in the r*Bs*HP2, r*Bs*GAL, r*Bs*FABP (*P* < 0.001), and r*Bs*HP1, r*Bs*UP, r*Bs*TLP (*P* < 0.01) vaccine groups were increased markedly; however, there was no difference in the r*Bs*GST and r*Bs*TPX groups compared with the adjuvant group (Fig. 5K). Likewise, a significant elevation of specific IgA was measured in mice immunized with r*Bs*HP2, r*Bs*HP1, r*Bs*UP, and r*Bs*GAL (*P* < 0.001), r*Bs*TLP and r*Bs*FABP (*P* = 0.003), and r*Bs*GST (*P* = 0.016), whereas the r*Bs*TPX (*P* = 0.99) group showed no difference compared with the adjuvant group (Fig. 5L).

### Cytokine levels

The larval burden in mice decreased after immunizing with the antigens, and an increase in antigen-specific antibodies was detected. The systemic cytokines in the sera were evaluated at 7 days after the second immunization to confirm the type of Th1/Th2 elicited by the candidate antigens.

**Fig. 6:**
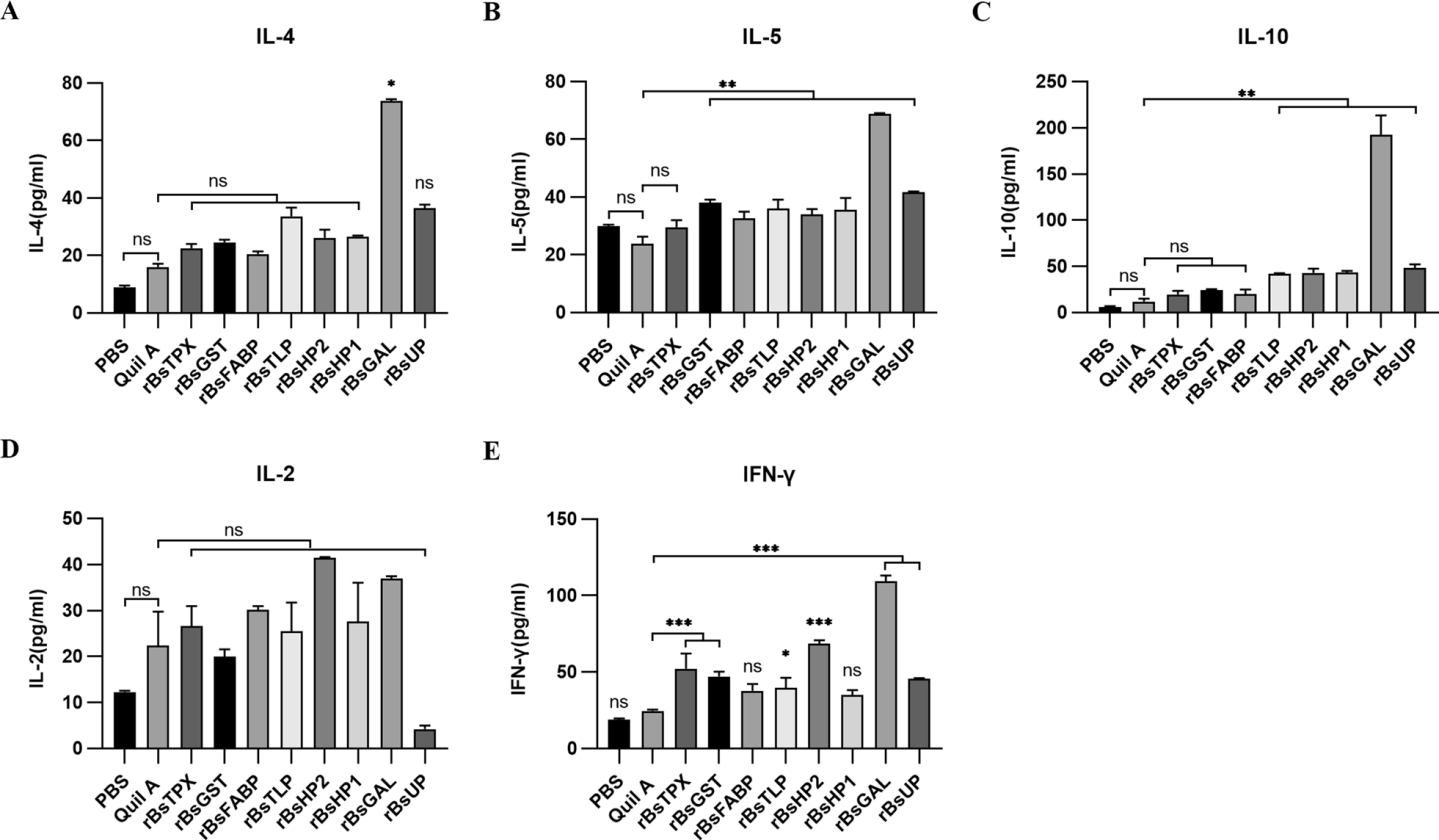
Systemic cytokine levels induced by *B. schroederi* antigens (r*Bs*TPX, r*Bs*GST, r*Bs*FABP, r*Bs*TLP, r*Bs*HP2, r*Bs*HP1, r*Bs*GAL, and r*Bs*UP) at 7 days the after the second immunization in mice. **A** IL-4, **B** IL-5, **C** IL-10, **D** IL-2, and **E** IFN-γ. (Treatment with adjuvant only formed the control group; significance analysis: ^*^P < 0.05, ^**^P < 0.01, ^***^P < 0.001)

Taking the adjuvant group as the control, immunization with the GAL antigen significantly increased the level of IL-4, while other antigens had no significant effect on IL-4 (Fig. 6A); the difference in IL-5 level after immunization with TPX was not significant, whereas significant IL-5 was produced in response to the other antigens (Fig. 6B). Antigens HP2, HP1, UP, GAL, and TLP induced an extremely significant increase in IL-10 levels (Fig. 6C). There was no significant difference in IL-2 production in response to the eight antigens (Fig. 6D). The different levels of IFN-γ after immunization with HP1 and FABP were not significant; however, IFN-γ levels were significantly elevated in response to the other antigens (Fig. 6E).

## Discussion

Recombinant subunit vaccines are easy to produce, cheap, and low-side-effect vaccines that use DNA recombination technology to produce the protective antigen of pathogens. These types of vaccines are safe and are the most widely used vaccines in humans and animals [35]. To date, recombinant subunit vaccines have been studied in nematodes, such as *A. suum*, *T. canis*, *Necator americanus*, *Haemonchus contortus*, and *Trichinella spiralis*, among which the recombinant vaccine against hookworm has entered the clinical trial stage [36]. Antigens with better immune protection effects have been screened from *A. suum* recombinant proteins, mainly including As16 [14], As24 [37], and As37, among which As37 might be a pan-worm candidate vaccine [38]. *T. canis * recombinant antigens rTcCad and rTcVcan reduced lung larvae by 54.3% and 53.9%, respectively, after immunization of mice [15], while rTcVcan formulated with Quil A adjuvant increased the protection rate of mice to 73% and also reduced 95% of the eggs in dog feces [39]. *T. spiralis * enzymes, such as TsAPP/TsCX and Ts-Adsp, can reduce adult worms by 63.9–71.1% and muscle larvae by 62.1–68.5% in mice after recombinant production and immunization [40, 41]. Vaccination with recombinant HcTTR and Hc32 for goats can result in the reduction of worm burdens [42, 43]. Canine hookworm recombinant antigens have been studied in different animal models [44], and immunization of animals with ASP [45], ASP-2 [46], GST [27], and CP-1 [47] reduced intestinal adult worm and fecal eggs counts. Phase 1 clinical trials of Ac-APR-1 and Ac-GST-1 have been carried out in Brazil, Africa, and the US [36, 48, 49]. A clinical trial of the combination of the two vaccines has been conducted in Gabon [50]. Therefore, the commercialization of the nematode recombinant subunit vaccine has good prospects.

Previous studies assessed the reduction rate of early larvae in the liver and lungs of mice at day 7 post-infection [18]. To comprehensively evaluate the protective effect of the vaccine against L3 larvae in the *B. schroederi*-infected mouse model, we chose day 20 post-infection to measure the reduction rate of L3 larvae and observe the pathological changes in the liver and lungs. This was based on the developmental time and main parasitic organs of the L3 larvae [8].

Previous studies showed that the candidate antigens of *B. schroederi* (PYP, Ag1, Ag2, and Ag3) displayed 62.9–71.2% protection after immunizing mice three times with Freund's adjuvant [13, 16–18]. By contrast, our antigens (HP2, HP1, GAL, and UP) formulated with Quil A provided and increased protection from 71.5% to 76.5% via a two-time injection in a mouse model. More importantly, we improved the protective effect of the vaccine and reduced the number of immunizations. Antigens combined with different adjuvants may cause different immune protection [38]. Totally, Quil A has been widely used in the veterinary field as a new-generation adjuvant with the characteristics of stimulating a long-lasting humoral and cellular immune response at low concentrations [39]. In this study, the Quil A mixed with recombinant antigen formulation could stimulate significant humoral and cellular immune responses. No side effects or allergies were observed in all mice during the experiment, consistent with our antigen allergy and toxicity prediction results. After screening the effective antigen, we will continue to investigate the cocktail vaccine for the screen of a better protective effect. To further verify the persistence and safety of the recombinant antigen with Quil A, the long-lasting antibody-level experiment of the vaccine and allergy study on guinea pigs will be conducted.

Parasite-secreted GST was involved in parasite survival, repair of host-induced damage, and modulation of host immunity [51] and showed better protective effects in *T. spiralis* [52], hookworms [48] and filarial worms [53]. In this study, the protective effect of GST was poor, which might be related to species differences. TPX, a member of the cysteine peroxidase-like family, protects worms from host reactive oxygen species [54]. Research on TPX candidate vaccines has been carried out in *T. spiralis* [29], filarial worms [55], and trematodes [56]. No protective effect was exhibited in the TPX group of *B. schroederi*, which might be related to the exposure of the epitope of TPX between species and interaction between antibodies. FABP plays a critical role in worm lipid transport, and is often used as a target to block lipid synthesis in parasites in vaccine research [57]. GAL is a lectin family member and participates in various key biological processes [58]. It can stimulate the host's immune response during parasite infection [59]. TLP belongs to the transthyretin-like family, is widely distributed in nematode excretion and secretion products, and has strong immunogenicity [60]. In this study, immunization with FABP, GAL, and TLP showed better protection, consistent with the good protective effect of FABP [28], GAL [26], and TLP [42] as candidate vaccine antigens in other parasites. Hypothetical proteins are conserved proteins that have been studied in parasites as candidate vaccines and drug targets [61, 62]. Excretory-secretory antigens have been validated as vaccine candidates in various nematodes [63]. At the same time, HP1, HP2, and UP are all highly expressed excreted/secreted antigens of unknown function [22], which are unique to *B. schroederi* and showed good protective effects in the mouse models. Some candidate antigens selected in this study have high sequence similarities with homologous antigens of other parasites but have shown differential protective effects in animal models [23–25]. Interestingly, we also found that the number of epitopes was not directly related to the protective effect of antigen. We speculated that the vaccine's protective effect might be associated with the conformation of the epitopes [64]. Therefore, the protective mechanisms and functions between different parasites are worthy of further study.

In this study, all antigens produced high levels of antigen-specific antibody IgG after immunization compared with that of the control group. Seven antigens (GST, FABP, TLP, HP2, HP1, GAL, and UP) induced a mixed antibody response after mouse immunization. Studies have shown that a mixed Th1/Th2 response might be more conducive to protective immunity than a polarized response [37, 38, 65].

IL-4 and IL-10 can regulate the damage caused by the Japanese roundworm larval migration when acting synergistically. IL-10 may participate in the control of inflammation, and IL-4 might promote tissue repair and wound healing by mediating macrophage and eosinophil responses [66, 67]. In this study, the levels of IL-10 were significantly increased when mice were immunized with HP2, HP1, UP, GAL, and TLP. At the same time, IL-4 only displayed a significant response to the GAL antigen compared with the adjuvant group, although IL-4 in the HP2, HP1, UP, and TLP groups did not reach a significant level; however, the levels were still higher than those of the other antigens. The scores for the surface lesions of the liver and lungs of the mice vaccinated with five antigens were lower. Eosinophils are important to regulate worm infection [33]. IL-5 is regarded as a potent regulator of formation, differentiation, maturation, activation, recruitment, and survival of eosinophils [68] and induces B cell differentiation and antibody secretion. After infection by worms, IL-5 can enhance eosinophil activation and kill larvae at the site of infection by releasing toxic eosinophil granules [69]. Nematode vaccine studies have shown a mixed Th1/Th2 immune response characterized by high levels of IFN-γ and IL-5 [37, 38, 65]. In the present study, the difference in IL-5 production after vaccination with TPX was not significant, and it also did not show protection; the other antigens produced significant IL-5 and significantly reduced the burden of larvae. Therefore, IL-5 might be an important cytokine against helminth infection. None of the eight vaccine groups induced significant IL-2, similar to the conclusion that the *A. suum* vaccine candidate did not induce IL-2; however, the specific mechanism remains unclear [33]. There was no significant difference in IFN-γ levels induced by HP1 and FABP, but there were significant differences for other antigens. IFN-γ activates macrophages and induces the production of nitric oxide synthase (iNOS), which has toxic effects on parasite metabolic processes [70]. The reduction of parasites was related to a mixed Th1/Th2 immune response, mainly characterized by high levels of specific antibodies (IgG, IgE, IgA, IgG1, and IgG2a) and significantly elevated cytokines (IFN-γ, IL-5, and IL-10).

## Conclusions

Eight prokaryotically expressed recombinant proteins (r*Bs*TPX, r*Bs*GST, r*Bs*FABP, r*Bs*TLP, r*Bs*HP2, r*Bs*HP1, r*Bs*GAL, and r*Bs*UP) were used to immunize mice (50 μg/mouse) twice, which were then challenged with *B. schroederi* eggs. The results showed that the worm reduction rates induced by r*Bs*HP2, r*Bs*GAL, and r*Bs*UP were 71.5%, 74.7%, and 76.5%, respectively. Moreover, the numbers and severity of liver and lung surface lesions were significantly decreased. The reduction of parasites and changes in liver and lung tissue lesions are related to a mixed Th1/Th2 immune response, as manifested by high levels of specific IgG, IgE, IgA, IgG1, and IgG2a and marked upregulation of cytokines IFN-γ, IL-10, and IL-5. The recombinant proteins r*Bs*HP2, r*Bs*GAL, and r*Bs*UP might be potential candidate vaccines against *B. schroederi* infection. Simultaneously, our study can provide a reference for the next step to study the protective effect of the cocktail vaccine in the mouse model.

## Data Availability

Molecular data have been deposited to GenBank with the following accession numbers: AJH66211.1, OQ263159-OQ263161, and OP807113—OP807116.

## Abbreviations

*Bs*: *Baylisascaris schroederi*
TPX: Thioredoxin peroxidase
GST: Glutathione S-transferase
FABP: Fatty acid-binding protein
TLP: Transthyretin-like protein 46
HP2: Hypothetical protein 2
HP1: Hypothetical protein 1
UP: Uncharacterized protein
GAL: Galectin
IgG: Immunoglobulin G
IgE: Immunoglobulin E
IgA: Immunoglobulin A
IFN-γ: Interferon gamma
IL: Interleukin
